# What Type of Social Support Is Important for Student Resilience During COVID-19? A Latent Profile Analysis

**DOI:** 10.3389/fpsyg.2021.646145

**Published:** 2021-06-22

**Authors:** Yingping Mai, Yenchun Jim Wu, Yanni Huang

**Affiliations:** ^1^Business School, Huaqiao University, Quanzhou, China; ^2^Graduate Institute of Global Business and Strategy, National Taiwan Normal University, Taipei, Taiwan; ^3^Leisure and Recreation Administration Department, Ming Chuan University, Taipei, Taiwan; ^4^Department of Psychology and Education, School of Shantou Polytechnic, Shantou, China

**Keywords:** social support, student resilience, coping style, latent profile analysis, mental health

## Abstract

In the face of the sudden outbreak of coronavirus 2019 (COVID-19), some students showed resilience in coping with difficulties while some did not. While different types of students showed different levels of resilience, are there significant characteristics among students with similar levels of resilience? In this study, 3,454 students (aged 15–25 years) were surveyed to understand students' perceived social support-coping modes while investigating the demographic characteristics and mental health status of subclasses of different modes. We found that (1) in the two subgroups of students with extremely low and low levels of perceived social support, the source of students' perceived social support did not have a clear orientation; in the two subgroups with moderate and high levels of perceived social support, the most perceived emotional support was from family and friends, while the least perceived support was companionship from teachers, classmates, and relatives, and problems related to the dependability of friends and communication with family. (2) The degree of social support perceived by students is directly proportional to the coping tendency, i.e., as the degree of perceived social support increases, the proportion of students adopting active coping strategies increases while that of students adopting negative coping strategies decreases; thus, we concluded that high levels of emotional support from family and friends can increase students' tendency of adopting positive strategies to cope with difficulties, while problems related to the dependability of friends and communication with family decrease students' tendency of adopting positive coping strategies. (3) Gender had a significant impact on the extremely low and low levels of perceived social support-negative coping tendencies; these subgroups accounted for 34.6% of the total students. Gender showed no significant influence on other subgroups, a school type had no impact on the distribution of the subgroups. (4) The higher the degree of perceived social support, the lower is the degree of students' general anxiety, and the lower is the degree of impact by the COVID-19 pandemic. The subdivision of student groups allows us to design more targeted support programmes for students with different psychological characteristics to help them alleviate stress during the COVID-19 epidemic.

## Introduction

Due to the sudden outbreak of coronavirus 2019 (COVID-19), comprehensive social distancing measures were widely adopted, and students had to shelter in place at home. Facing stress caused by various difficulties, some students showed resilience, such as adopting active countermeasures as well as a more stable psychological state to deal with the crisis, while some students did not. Based on social support theory, this study aims to explore the impact of social support on students' resilience. Specifically, this study will discriminate the different perceived levels of social support among students and its impacts on students' coping tendency, and then further investigate the effect of social support-coping mode on students' mental health. The results of previous social support theory-related studies show that social support, as an important environmental resource for individuals in social life, is closely related to the control and prevention of negative emotions; good social support can provide protection for individuals under stress and has a generally beneficial effect on maintaining the health and stabilizing the mood of individuals (Martín-Albo et al., 2015; Hou et al., 2020). The same stressful situation can have different impacts on different individuals (Guay et al., 2013). Generally, those who receive more support from family or friends have a stronger mental capacity and are more mentally and physically healthy (Seiffge-Krenke and Pakalniskiene, 2011; Cao et al., 2020); in contrast, those who rarely receive similar support have a low mental capacity and poor mental and physical health (Elmer et al., 2020; Li et al., 2020). In other words, an individual's social relationship background had positive effects on his/her resistance, mitigation as well as prevention, and had a beneficial bearing on his/her health (Haber et al., 2007; Stenling et al., 2015; Labrague et al., 2020).

Social support refers to the care and support people feel from others (Raschke, 1977). Based on the perspective of interpersonal relationships, social support can be divided into four categories: emotional support refers to providing others with empathy, warmth, love, and trust; instrumental support refers to providing material help and services when others are in need; informational support refers to helping others solve problems by providing useful suggestions, information, etc.; and appraisal support refers to providing useful information for others' self-evaluation (House et al., 1988; Taylor et al., 2007). Haber et al. (2007) hold that social support is a broad term encompassing a variety of constructs but it can be divided into two types: (1) objective and visible support, including material support, network support (stable social relationships such as marriage, colleagues, friends, etc., as well as unstable social contacts such as informal groups, etc.), which is not dependent on an individual's perception and thus an objective reality, and (2) subjective support, i.e., emotional support and feelings of respect and understanding by an individual in social life, which are closely related to the individual's subjective feelings. Therefore, social support can be viewed as subjective support through various social relationships based on the social network of an individual or its objective impact on the individual. By helping an individual cope with and recover from difficult situations and adversity and improving an individual's positive mental state, social support plays a critical role in an individual's mental resilience (Cao et al., 2020; Yildirim and Tanriverdi, 2020). Studies have shown that peer support, which is the interpersonal connections among age-matched individuals established in common activities and mutual cooperation, is an important source of social support for college students (Lamis et al., 2016; Burns et al., 2020). When an individual is under pressure or feeling threatened, these connections provide an individual moral and material resources to help the individual relieve stress, reduce stress-induced negative emotions, and thus become an important factor affecting the individual's adaptation to the adverse situation. For college students who are away from families, schoolmates, friends, and other peers may be the most trustworthy members of their social networks and a particularly important source of social support. Studies have also shown that family support can effectively help students cope with difficult situations, reduce mental stress, and prevent mental health problems, thereby maintaining their mental health development (Chang et al., 2020). Certainly, other sources of social support, such as relatives, teachers, etc., can also have a general beneficial effect on the intention of students' risk taking as well as their physical and mental health improvement (Liu et al., 2019; Zhou, 2020).

Previous studies of college students' social support have addressed students' demographic characteristics and the effect of social support on an individual's mental health problems, e.g., the degree of social support obtained by male students is significantly higher than that by female students (Elmer et al., 2020), and the mental health effects of social support are directly correlated with the personality characteristics of the supported person (Liu et al., 2020). Regardless of findings, these studies overlook the internal heterogeneity of college students when perceiving social support. According to positive psychology, in particular studies on psychological resilience, relative to differences between different groups, the differences in the mental health of individuals in disadvantaged situations are more worthy of attention. Friborg et al. (2006) found that individuals with high levels of psychological resilience perceive less mental distress and show higher levels of mental health than those with low levels of psychological resilience and that psychological resilience has a moderating effect on stress and mental distress (Friborg et al., 2006; Ratelle et al., 2013). Therefore, understanding the heterogeneity within groups of students who perceive social support and adopt corresponding coping strategies differently allow distinguishing the effects of different sources of social support on students when coping with difficulties. On this basis, the in-depth investigation of subgroups of different modes can be conducted to formulate more effective and more targeted programmes to help students through the COVID-19 pandemic and similar difficulties. In this study, we examined students' social support perception-coping tendencies by subdividing the coping tendency heterogeneity on the basis of perceived social support and demographic characteristics of student subgroups with various perception-coping categories and their mental health status.

In recent years, latent profile analysis (LPA) has been widely applied for the classification of heterogeneous groups in many fields such as sociology, biomedicine, and psychology. It can be used to simulate the likelihood (i.e., probability) that an individual belongs to a personality profile (Wang and Hanges, 2011), i.e., with the highest intragroup similarity within a subgroup and the lowest similarity in different subgroups, enabling the examination of students' perceived social support-coping strategies more accurately while providing criteria to help investigators determine the best solutions. Researchers can identify unobserved heterogeneity in LPA sample data through modeling (Meyer and Morin, 2016; Morin et al., 2016; Wang et al., 2016). After determining the solution for a profile, it is possible to predict the members of a profile using antecedent variables, i.e., if a high/low measurement for a member for one antecedent variable increases or decreases perception, the member may belong to a specific profile aspect (Vermunt, 2010; Gabriel et al., 2015).

Built on the previous researches and methodologies, in this study, we conducted LPA based on three common forms of social support to understand the overall perceived social support model of the participants and then divided the participants into two subgroups (tendency of adopting positive coping strategies and tendency of adopting negative coping strategies) to construct an LPA model to classify subgroups in terms of perceived social support-coping tendencies; furthermore, we examined the coping strategies of secondary technical school students and college students under each social support mode during the COVID-19 pandemic and the major demographic characteristics of the students. By taking full account of the internal heterogeneity within a subgroup, using LPA, we further investigated various internal problems (e.g., anxiety and stress-induced trauma) related to different perceived social support-coping tendencies and their differences.

## Materials and Methods

### Participants

We conducted an online questionnaire survey of students, recruited using the cluster sampling method, from four campuses of a vocational school in Shantou City, China. Before sampling, we obtained the school's support, and each participant signed an informed consent form prior to beginning the questionnaire; we asked the psychological counseling center of the school to issue the questionnaire. The survey was conducted from February 2–6, 2020, during which 5,021 questionnaires were collected, from which 3,454 valid samples were obtained after screening. Among the respondents, 3,416 (98.9%) were students staying in Guangdong province, 44 majors included. Thousand two-hundred and sixty-five were students from secondary technical school, of which 423 (33.4%) were male students and 842 (66.6%) female students, and 2,189 were junior college students, of which 782 (35.7%) male students and 1,407 (64.3%) female students. Male and female students took up 36.5 and 63.5% of the total valid survey population, respectively. The respondents were 15–25 years of age (mean age: 20.03; *SD:* 1.734).

### Tools

We used four questionnaires to measure four dimensions (perceived social support, coping tendency, generalized anxiety, and stress reaction) and collected the respondents' sociodemographic information, including gender, age, grade, place of residence, education level, etc.

#### Perception of Social Support Scale

The Perception of Social Support Scale (PSSS) assesses an individual's self-understanding and self-perception and reflects the individual's overall level of perceived social support. Formulated by Zimet et al. (1990) and introduced and revised by Qianjin Jiang (Zimet et al., 1990), the scale includes three dimensions, i.e., family support, friend support, and other support, and each dimension contains four items, for a total of 12 items, and is scored using an 7-point Likert scale; the higher the score is, the higher the perceived social support. The Cronbach's alpha value for the questionnaire used in this study is 0.942.

#### Simplified Coping Style Questionnaire

Coping style was measured using the Simplified Coping Style Questionnaire (SCSQ). This questionnaire was developed by Xie based on the Ways of Coping Questionnaire by Folkman and Lazarus (1988) (Xie, 1998). It is a 20-item self-report questionnaire that includes two dimensions, active coping (12 items) and passive coping (8 items), with higher scores representing greater active/passive coping. Participants are asked to agree or disagree, using a 4-point Likert scale (from 1 “never” to 4“very often”), according to how frequently they adopt each item. The instrument has been commonly used in China, and the Cronbach's alpha coefficient is 0.888.

#### Generalized Anxiety Disorder

The scale Generalized Anxiety Disorder (GAD-7) includes seven items to assess an individual's anxiety symptoms in the past 2 weeks and is scored using a 4-point Likert scale (0 = Never; 1 = Once in a few days; 2 = Once in more than half of the days; 3 = Once almost every day), with a total score ranging from 0 to 21 (Spitzer et al., 2006); the higher the score is, the more severe the anxiety: 0–4 points, normal; 5–9 points, mild anxiety; 10–14 points, moderate anxiety; and 15–21 points, severe anxiety. The Cronbach's alpha value for this questionnaire is 0.930.

#### Impact of Events Scale

The Impact of Events Scale (IES-6) is simplified from the Impact of Events scale (IES-R) by Thoresen and thus is highly correlated with the ISE-R (Thoresen et al., 2010). It is a powerful and simplified version of the ISE-R, contains six items in three dimensions [intrusion symptoms (4–5), avoidance symptoms (1, 3), and high alertness (2, 6)] and is scored using a 5-point Likert scale (0 = Never; 1 = Occasionally; 2 = Sometimes; 3 = Frequently; and 4 = Always). The average score (total score divided by 6) is used to assess the likelihood of PTSD: <1.09, normal; 1.09 ≤ and <1.5, likely PTSD; ≥1.5, PTSD. The Cronbach's alpha value for this questionnaire is 0.803.

### Statistical Analysis

We used Mplus 8.3 to perform LPA of the overall data to examine the difference in the levels of perceived social support in the tested groups. Using SPSS 19.0, we calculated the mean and standard deviation for the scores for the 20 coping strategy items; the data were then Z-transformed to be standardized and worked out the coping tendency score. Based on the coping tendency score, we divided the participants into two groups, i.e., those who adopted positive coping strategies (positive coping group) and those who adopted negative coping strategies (negative coping group), to compare the differences in individuals with different levels of perceived social support. Furthermore, we examined the differences in demographic characteristics within each subgroup through multivariate logistics regression analysis using the secondary subdivision result as the dependent variable and other indicators (e.g., gender and age) as independent variables. Finally, we compared the mental health differences among individuals in each subgroup through single factor analysis of variance (ANOVA).

## Results

### LPA of Students' Perception of Social Support

To examine perceived social support patterns among secondary technical school students and junior college students, we performed LPA of the PSSS scores. The fit indices for two to five classes were extracted and analyzed with model comparison. The model fit test indicators included the Akaike information criterion (AIC) and Bayesian information criterion (BIC), the sample size-adjusted BIC (aBIC), and entropy index (Xian et al., 2005), the likelihood ratio test indicator Lo-Mendell-Rubin adjusted likelihood ratio test (LMR-LRT) (Lo et al., 2001), and the bootstrap-based likelihood ratio test (BLRT) index (Peel and McLachlan, 2000). The lower the values are for the three information assessment indicators, the better the model fit. The valuation range for the entropy index is 0–1, and the closer the value is to 1, the more accurate the classification; entropy <0.60 indicates that more than 20% of individuals are misclassified, and entropy = 0.8 indicates that the accuracy of the classification exceeds 90%. If the *p*-values for the LMR-LRT and BLRT reach a statistically significant level, then the K-class model is significantly better than the k-1-class model (Muthén and Muthén, 2000).

The data showed that the conclusions based on the different information indicators were inconsistent and that the entropy values for the four models all exceeded 0.8. When five classes were retained, the AIC, BIC, and aBIC values were low, and the LMR-LRT(*p*) and BLRT(*p*) values were lower than 0.01, indicating that in terms of the indicators, all four latent class models satisfied statistical requirements. The estimated condition means for the four latent class models of the 12 items of perceived social support are shown in Table 1.

**Table 1:** Latent profile classification of the perceived social support of the overall population.

**Index**	**Number of model**			
**Total (*n* = 3,454)**	**2**	**3**	**4**	**5**
AIC	128,229.515	124,371.861	120,824.736	118,358.292
BIC	128,456.964	124,679.225	121,212.015	118,825.485
aBIC	128,339.398	124,520.351	121,011.835	118,583.997
Entropy	0.952	0.972	0.930	0.958
LMR-LRT(*p*)	<0.001	<0.001	<0.001	<0.001
BLRT(*p*)	<0.001	<0.001	<0.001	<0.001

**Figure 1:**
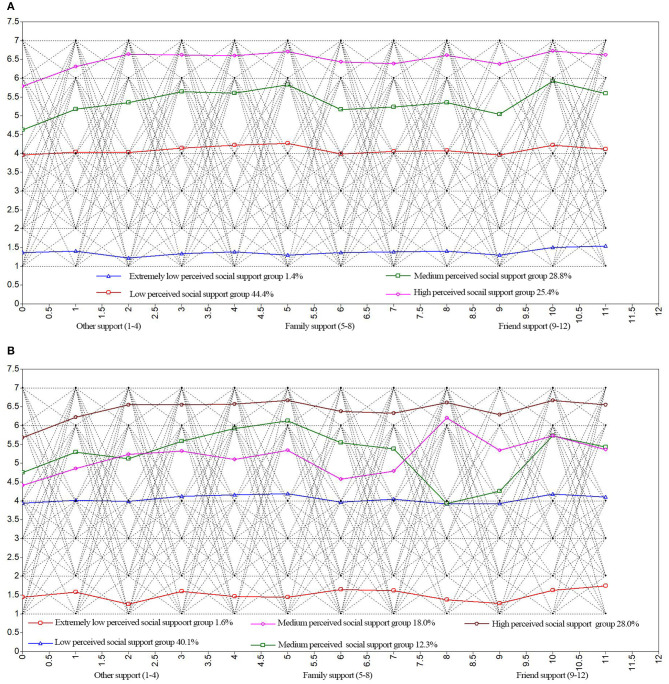
**(A)** Four and **(B)** Five classes of perceived social support of the overall population. ELSSPG, Extremely low perceived social support group; LPSSG, Low perceived social support group; MPSSG, Medium perceived social support group; HPSSG, High perceived social support group.

In terms of statistical indicators, with each added subclass, the AIC, BIC, and aBIC values decreased. However, when determining the best model, the interpretability of each class should also be considered (Finch and Bronk, 2011). Figures 1A,B show that when four and five classes, respectively, are retained, the conditional probabilities of the extremely low perceived social support group, low perceived social support group and high perceived social support group were very similar. However, in the five-class model, two classes were intertwined with each other at the medium perception level, with non-significantly different conditional probabilities and a weak explanatory power. Nevertheless, the four-class model classified each class very well, with an entropy value of 0.930, indicating that the four-class classification was accurate; therefore, this model was chosen as the optimal model for this study after comprehensive consideration.

Figure 1A shows that the conditional mean values for the four latent classes of the 12 items in the three perceived social support dimensions were significantly different, indicating different characteristics. Among them, for Class 1 (C1), the conditional mean values for the three dimensions of perceived social support were all the lowest, showing no significant fluctuations; C1 contained a total of 50 individuals, accounting for 1.4% of all participants, and based on their scores, C1 was defined as the “extremely low perceived social support group (ELPSSG).” The overall scoring trend for Class 2 (C2) was similar to that for C1 but with significantly higher conditional mean values; therefore, C2 was defined as the “low perceived social support group (LPSSG),” containing 1,534 individuals, accounting for 44.4% of the total participants. The conditional mean values for Class 3 (C3) were higher than those for C2; therefore, C3 was defined as the “medium perceived social support group (MPSSG),” containing 985 individuals, accounting for 28.8% of the total participants. The scores for individuals in Class 4 were the highest among all groups for each item; therefore, C4 was defined as the “high perceived social support group (HPSSG),” containing a total of 885 individuals, accounting for 25.4% of the total participants.

The scores for the ELPSSG and LPSSG did not differ significantly for all items, indicating that the classification of social support sources perceived by these two groups is unclear. The MPSSG recorded low scores for “when I have difficulties, someone (teacher, classmate, or relative) will show up,” “when in trouble, I can rely on my friends,” and “I can talk about my problems with my family” but high scores for “I get emotional help and support from my family when in need” and “my friends can share both happy and sad times with me,” indicating that individuals in this class can clearly determine the type of social support source they perceive. The perception trend for the HPSSG group was similar to that for the MPSSG group but with smaller differences between the classes. In other words, emotional support from family and empathy with friends were the most obvious perceived sources of social support, while the support source of solving practical difficulties was less perceived by the students.

### Analysis of Coping Tendencies for Different Perception Classes

Based on the four latent classes from the LPA, we examined the relationship between students' perceived social support and their coping tendencies when facing the COVID-19 pandemic. First, through the SCSQ, we divided the overall population into two groups: positive coping group and negative coping group. Specifically, using SPSS 19.0, we calculated the mean and standard deviation for the scores for the 20 coping strategy items; the data were then Z-transformed. Based on the formula for coping tendency, i.e., coping tendency = (total score for the coping strategy items – mean)/standard deviation, to obtain the Z-standardized score for each coping strategy, the value for coping tendency was obtained by subtracting the standardized negative coping strategy score from the standardized positive coping strategy score. If the coping tendency value was negative, the participant mostly adopted negative coping strategies under stress; if the coping tendency value was 0 or positive, the participant was more inclined to adopt positive coping strategies under stress. Accordingly, the individuals were divided into two groups: positive coping group and negative coping group. Combining the LPA analysis results for the four latent classes and the two coping tendency groups, we generated eight subclasses and further examined their differences and trends. The subdivision results are provided in Table 2.

**Table 2:** Subdivided classes of perceived degree of social support-coping tendency.

**Latent profile**	**Coping style**		
	**Negative *n* (%)**	**Positive *n* (%)**	**Total (*n*)**
ELPSSG	43 (86%)	7 (14%)	50
LPSSG	1,151 (75%)	383 (25%)	1,534
MPSSG	541 (54.9%)	444 (45.1%)	985
HPSSG	383 (43.3%)	502 (56.7%)	885
Total (*n*)	2,122	1,332	3,454

*ELSSPG, Extremely low perceived social support group; LPSSG, Low perceived social support group; MPSSG, Medium perceived social support group; HPSSG, High perceived social support group*.

**Figure 2:**
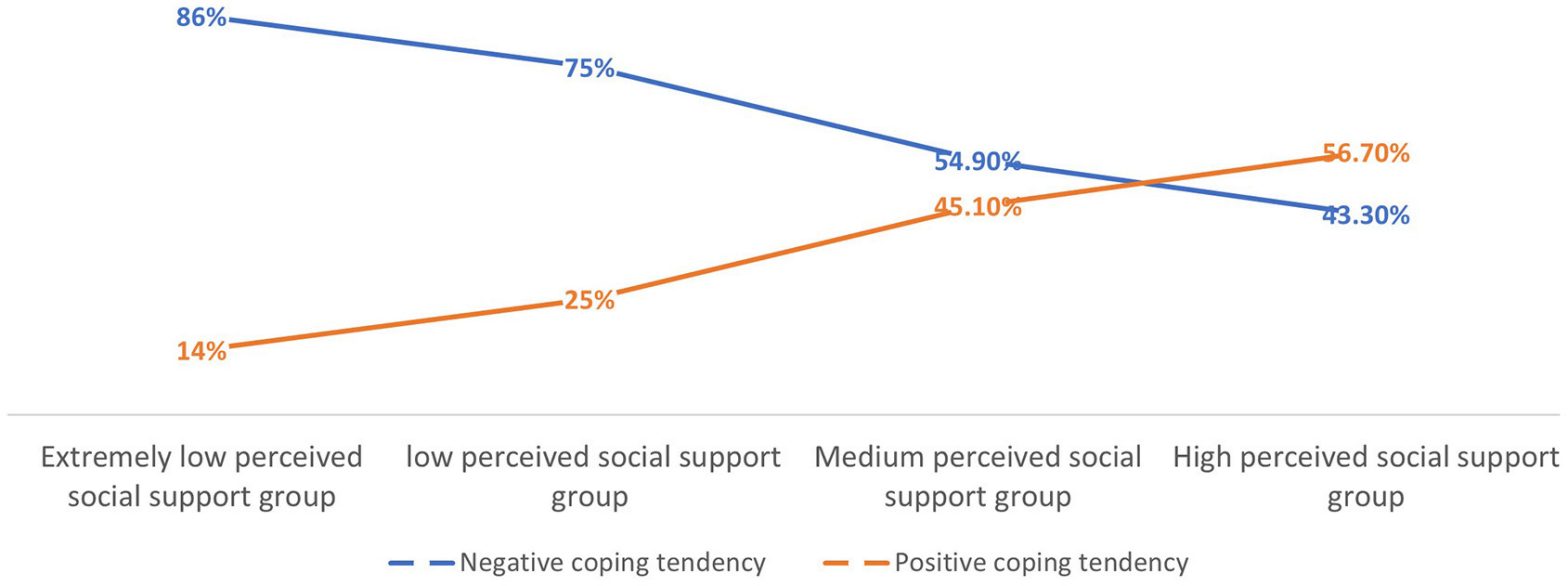
Trends for subclasses of different perceived levels of social support-coping styles.

We named the eight subclasses according to the perceived level of social support-coping style as follows: ELPSSG-negative coping style (ELPSSG-N): 43 individuals, accounting for 86% of the total participants; ELPSSG-positive coping style (ELPSSG-P): 7 individuals, accounting for 14% of the total participants; LPSSG-N: 1,151 individuals, accounting for 75% of the total participants; LPSSG-P: 383 individuals, accounting for 25% of the total participants; MPSSG-N: 541 individuals, accounting for 54.9% of the total participants; MPSSG-P: 444 individuals, accounting for 45.1% of the total participants; HPSSG-N: 383 individuals, accounting for 43.3% of the total participants; and HPSSG-P: 502 individuals, accounting for 56.7% of the total participants. The trends for the subclasses are provided in Figure 2.

The results shown in Table 2 and Figure 2 indicate that with an increased level of perceived social support, the proportion of individuals who adopted negative coping strategies in the negative coping tendency group declined; in contrast, the proportion of individuals who adopted positive coping strategies in the positive coping tendency group increased. The level of perceived social support is positively correlated with coping tendency.

### Analysis of Gender and Grade for Different Perception Classes

Based on the LPA results, we further examined the demographic characteristics of each social support perception class. We conducted a multivariate logistic regression analysis using the LPA results as the dependent variable and gender (females as the reference) and grade (junior college as the reference) as independent variables, with Class HPSSG-P as the reference class for comparison. The odds ratio (OR) was obtained to reflect the ratio of the ratios of gender to grade at each level of perceived social support. The multivariate logistic regression analysis results are provided in Table 3.

**Table 3:** Latent profile and coping tendencies for different levels of perceived social support.

		**Gender**		**School type**	
**Subgroup**	**OR/CI**	**Female**	**Male**	**College**	**Secondary school**
ELPSSG-N	OR	1.00	5.049^**^	1.00	0.623
	CI (95%)		2.592–9.834		0.306–1.268
LPSSG-N	OR	1.00	1.789^**^	1.00	1.609
	CI (95%)		1.428–2.243		0.860–1.330
MPSSG-N	OR	1.00	1.072	1.00	1.040
	CI (95%)		0.821–1.399		0.809–1.338
HPSSG-N	OR	1.00	1.220	1.00	0.953
	CI (95%)		0.915–1.626		0.772–1.258
ELPSSG-P	OR	1.00	3.306	1.00	1.345
	CI (95%)		0.730–14.964		0.297–6.084
LPSSG-P	OR	1.00	0.988	1.00	1.145
	CI (95%)		0.784–1.245		0.919–1.427
MPSSG-P	OR	1.00	1.264	1.00	0.965
	CI (95%)		0.949–1.683		0.731–1.273
HPSSG-P	OR	1.00	0.933	1.00	0.990
	CI (95%)		0.702–1.239		0.759–1.291

*ELSSPG, Extremely low perceived social support group; LPSSG, Low perceived social support group; MPSSG, Medium perceived social support group; HPSSG, High perceived social support group; N, Negative coping tendency; P, Positive coping tendency*.

** *means p < 0.01*.

HPSSG-P, as the reference group, was compared with the other seven subclasses, and the ORs showed that the population distribution for the coping tendency-social support model was not significantly affected by grade. Two classes (ELPSSG-N and LPSSG-N) were significantly affected by gender, but for all the other classes, gender showed no impact. Therefore, most of the time, gender was not an important factor as it only worked in extremely negative situations, such as low perceived social support and negative with negative coping tendency.

### Comparisons for Students' Coping Tendency-Perceived Social Support

The ANOVA results for the anxiety scores for different coping tendency-perceived social support latent classes, *F* = 30.768 (*P* < 0.01), and for the standardized COVID-19 influence scores, *F* = 6.177 (*P* < 0.01), are provided in Table 4. Multiple comparisons showed that in terms of generalized anxiety, the scores for all the positive coping subgroups were lower than those for all the negative coping subgroups, indicating that the level of anxiety in individuals in the positive coping group was lower than that in individuals in the negative coping group. More importantly, as the level of perceived social support increased, the level of anxiety in individuals with negative coping tendencies decreased, but in individuals with positive coping tendencies, the level of anxiety increased first and then declined. In terms of the impact of the COVID-19 pandemic, the scores for all the positive coping subgroups were lower than those for all the negative coping subgroups, but those for the individuals in the ELPSSG were higher. Notably, the score for the negative tendency group fluctuated significantly, while that for the positive tendency group was low but increased. Overall, students' mental health was better in the higher perceived social support− positive tendency groups, than the lower ones.

**Table 4:** Impact of the COVID-19 pandemic and anxiety levels in different latent profile.

	**Negative groups (*****M*** **±*****SD*****)**				**Positive groups (*****M*** **±*****SD*****)**				
	**ELPSSG-N**	**LPSSG-N**	**MPSSG-N**	**HPSSG-N**	**ELPSSG-P**	**LPSSG-P**	**MPSSG-P**	**HPSSG-P**	***F***
	**(*N* = 43)**	**(*N* = 1,151)**	**(*N* = 541)**	**(*N* = 383)**	**(*N* = 7)**	**(*N* = 383)**	**(*N* = 444)**	**(*N* = 502)**	
GAD	3.35 ± 5.136	3.02 ± 3.808	2.55 ± 3.106	2.13 ± 2.850	0.29 ± 0.488	1.36 ± 2.633	1.45 ± 2.321	1.09 ± 1.992	30.768
IES	0.957 ± 0.781	1.246 ± 0.698	1.199 ± 0.602	1.230 ± 0.648	1.095 ± 0.838	1.088 ± 0.633	1.083 ± 0.681	1.113 ± 0.613	6.177

*GAD, Generalized anxiety disorder, IES, Impact of Events Scale, ELSSPG, Extremely low perceived social support group; LPSSG, Low perceived social support group; MPSSG, Medium perceived social support group; HPSSG, High perceived social support group; N, Negative coping tendency; P, Positive coping tendency*.

## Discussion

COVID-19 has had a globally devastating impact, threatening not only the learning of tens of millions of students but also their mental health. The impact of such public health emergencies is destructive, unpredictable and overwhelming and that their impact on an individual's mental health may manifest as acute mental stress as well as chronic mental stress and corresponding mental reactions. The same is true for secondary technical school students and college students (Grubic et al., 2020). Due to the spread of the epidemic, a host of factors, such as stress regarding health, increased time living with parents, changes in learning style and environment, etc., have caused students to be unable to perform their duties. For students, the effect of deferred school openings, decreased employment opportunities, and school transfers has caused tremendous pressure. According to the results of data analysis, the coping ability of college students was shown to be at an intermediate level, a result that might be related to the fact that college students had trouble adapting to the epidemic, academic arrangements, and other factors. Therefore, understanding the perceived social support patterns and coping tendencies of the student population will help build a more targeted social support system to help them cope with the COVID-19 pandemic and similar crises.

In this study, we found that 50 students in the ELPSSG (accounting for 1.4% of the total participants) only perceived extremely low social support, while 1,534 students in the LPSSG (accounting for 44.4% of the total participants) perceived a medium level of social support (4 points); students in both groups were unable to clearly perceive the source of social support they received. In addition, 985 students in the MPSSG (accounting for 28.8% of the total participants) perceived moderate emotional support and lower levels of substantial support from family and friends, e.g., when in trouble, the companionship of teachers, classmates, or relatives; the dependability of friends; and communication with family were at a medium level of perception. Last, 885 students in the HPSSG (accounting for 25.4% of the total participants) perceived a high degree of overall support. Relative to those in the MPSSG, students in the HPSSG perceived lower companionship of teachers, friends, or relatives, with higher dependability of friends and few communication problems with their family. Overall, emotional support from family and friends enabled the students to perceive a high level of social support; however, the dependability of friends and communication problems with family reduced students' perceived social support, this result is consistent with the suggestion of Ratelle et al. (2013), the higher of perceived social support, the higher of positive attitude. To conclude, individuals were able to clearly judge the type and degree of social support sources as the key factor which allows the determination of favorable and unfavorable factors in students' social support system.

We also examined the effect of different levels of perceived social support on students' coping tendencies and found that the level of perceived social support is positively correlated with students' coping tendencies. The higher the perceived social support level, the higher is positive coping tendencies and the lower is negative coping tendencies. If students cannot clearly identify types of social support sources, they are more inclined to adopt negative coping strategies. For example, in the ELPSSG and LPSSG, the students had similar scores for each item but no distinct source of social support, and 86 and 75% of the students, respectively, were inclined to adopt negative coping strategies. Individuals who can clearly perceive the emotional support of family and friends have a reduced probability of adopting negative coping tendencies, e.g., the probability of individuals in the MPSSG and HPSSG adopting negative coping strategies decreased to 54.9%. With the increased dependability of friends and decreased communication problems with family, the probability of students adopting negative coping strategies decreased further to 43.3%, as in the case of the HPSSG. These results again verified the hypothesis of the social support theory proposed, i.e., support from social relations can prompt individuals to adopt positive coping strategies (Andrews et al., 1978), the family's role of emotional support and friends' emotional support (Labrague et al., 2020; Liu et al., 2020). The results indicate that in stressful situations, those who have a high level of physical or mental support from a spouse, friend or family member have better mental and physical health than those who have a low level of physical or mental support.

In contrast to the finding of Elmer et al. (2020), female students appeared to have worse mental health trajectories when controlling for different levels of social integration and COVID-19 related stressors, our research showed partial different results, by examining the demographic characteristics of participants in the eight subclasses, we found that two subclasses, ELPSSG-N and LPSSG-N, with a total of 1,194 individuals (34.6%), were significantly impacted by gender, while the other six subclasses were not, a result that is consistent with those from other studies. Furthermore, secondary technical school students and junior college students were assessed separately regarding the effect of grade on students' perceived social support-coping strategy. Grade had a non-significant impact on students in all subclasses, indicating that, for 15–25 year-old students in secondary technical school or junior college, their perceived social support-coping patterns are the same and that the results of this study have a wider application range.

Social support can benefit individuals' mental health, and those with a high level of social support have more emotional stability and better physical and mental health than those with a low level of social support (Mak et al., 2011; Cao et al., 2020). We examined the mental health status of participants in eight latent classes of perceived social support-coping strategies using the IES-6 and GAD-7. We found that in terms of the impact of the COVID-19 pandemic, the scores for all groups with positive coping tendencies were lower than those of groups with negative coping tendencies; however, those for individuals in the ELPSSG (1.5%) were higher. These findings verified, to a certain extent, that the degree of perceived social support-coping tendency is positively correlated with students' emotional stress response. Except for the outlier subclass ELPSSG, the other six subclasses showed the high score initially and then a low score. These findings verified the hypothesis that social support has a universal beneficial effect on individuals, it can help the individual cope with the stress and improve their mental health. The general effectiveness of social support may be derived from stable social network providing positive experiences. For example, an individual who obtains help and support from others has reduced anxiety and fear when facing a stressful event; when facing a stressful event again, the individual can better cope with stress and improve his/her mental health thanks to previous experiences and existing social support. Moreover, a stable social network also improves an individual's sense of self-worth while avoiding negative experiences (Grubic et al., 2020; Li et al., 2020; Yildirim and Tanriverdi, 2020). Thus, our findings support the viewpoints of Cao et al. (2020) and Mak et al. (2011), social support has a buffering effect on individuals facing stress and can mitigate the negative impact of stress events on physical and mental health and thus maintains and improves physical and mental health.

The level of anxiety in individuals who adopted positive coping strategies was lower than that of those who adopted negative coping strategies. The level of anxiety in individuals with negative coping tendencies decreased as perceived social support levels increased, while that in those who adopted positive coping strategies increased first and then decreased. These trends were likely due to the in ability of the students to clearly perceive social support sources and, thus, impossible for them to know whether the positive actions they take are supported. Moreover, anxiety may also be related to the contradiction between the high level of perceived emotional support from family and friends and low friend dependability and decreased family communication. The anxiety level of participants in the MPSSG-P subclass was significantly lower than that of participants in the HPSSG-P subclass; the conflict among these factors was more substantial in the MPSSG-P than in the HPSSG-P. The results of this study partially verify that social support has a positive effect on health, can buffer the impact of stress and provides emotional support and instrumental support so that individuals can better adapt to stress to improve health (Martín-Albo et al., 2015; Stenling et al., 2015), as well as support the findings of Yan and Zheng (2003), social support has a significant regression effect on individuals' subjective well-being as well as a positive correlation with the total score for subjective well-being, life satisfaction, and positive emotions and a negative correlation with negative emotions (Yan and Zheng, 2003; Chang et al., 2020). Therefore, support from social relations can enhance individuals' ability to regulate emotions by prompting them to adopt positive coping strategies, thereby obtaining a higher level of subjective well-being.

The results of our findings offer some practical implications. Firstly, according to our findings, emotional supports from family is important to students' resilience. We suggest that this kind of social support should be promoted by improving the parent-child communication. For example, the relevant parties should be frank with each other and talk with each other about their stress, anxiety, and uneasiness to gain and enhance mutual understanding and support. Family members should keep in touch with each other and help, support and encourage each other so as to strengthen family cohesion and form family dependence. Secondly, the findings showed that the support from teachers was insufficient due to the possible reason that students stayed at home in the period. The possible idea may be by leveraging social medias, teachers may deliver their support to students more effectively (Wu and Song, 2019). For example, through online teaching, training, classes, themed activities, and psychological consultations, with the school as the center and activities as support, teachers should guide and help students to adapt to new learning methods and establish channels for communication so that students can receive academic assistance and enhance their spiritual identity. Finally, students might interact with peers such as communication, exchange, and discussion with classmates by participating in learning activities and sharing interests and issues with friends may obtain understanding, support, trust, and companionship.

### Limitations and Future Research Directions

As with any empirical study, this work cannot avoid its limitations. First, although we tried our best to expand the sample size, the bias of sample selection such as all the students coming from the same province may limit the generalization of the findings. Besides, the findings are based on self-reported data and may constraint the reliability of the result. Future research might consider in-depth interviews to provide triangulated information as complementary illustrations of the findings. Furthermore, this paper offers insights on emotional supports from family and friends that affected student's resilience during the COVID-19 pandemic. Future investigations should experiment on explaining why and how these two types of social support sources can stimulate students' positive coping tendency and thus improve their mental health.

Second, our work focused on the role of students' perception of social support on resilience in the context-specific of the Covid-19 pandemic. Future work might verify if the findings can be applied to other contexts or not. For example, future work might compare the role of students' perception of social support on resilience between ordinary difficulty and extreme crisis. In addition, this work focused on students' perception of family support, friend support, and other support. Future research might explore whether there are other factors that affect the positive tendency response of students.

## Conclusion

In summary, this paper aims to explore the impact of the perception of social support and students' resilience of students from secondary technical school and college (15–25 years old) on their ability to cope with the impact of the COVID-19 pandemic and stress. Our results show that (1) students' inability to clearly perceive social support sources reduces their tendency of adopting positive coping strategies while students are more likely to take positive coping strategies when they perceived higher level of social support; (2) individuals with a high level of perceived emotional support from family and friends can increase their tendency of adopting positive coping strategies, while a low level of perceived help from teachers, classmates and relatives, a lack of dependable friends, and communication problems with family will reduce students' tendency of adopting positive coping strategies; (3) The higher the degree of perceived social support the more likely students adopt positive coping strategies, and thus improve their mental health. The findings are conducive to designing more targeted support programmes for secondary technical school students and college students to alleviate their stress caused by the COVID-19 pandemic.

## Data Availability Statement

The raw data supporting the conclusions of this article will be made available by the authors, without undue reservation.

## Author Contributions

YM carried out the concepts, design, data analysis, and manuscript editing. YW carried out the concepts, design, and manuscript editing. YH carried out the data acquisition and manuscript editing. All authors contributed to the article and approved the submitted version.

## Conflict of Interest

The authors declare that the research was conducted in the absence of any commercial or financial relationships that could be construed as a potential conflict of interest.
